# The Impact of Pretrained Language Models on Negation and Speculation Detection in Cross-Lingual Medical Text: Comparative Study

**DOI:** 10.2196/18953

**Published:** 2020-12-03

**Authors:** Renzo Rivera Zavala, Paloma Martinez

**Affiliations:** 1 Department of Computer Science and Engineering Carlos III University of Madrid Madrid Spain; 2 Department of Computer Science and Engineering Universidad Católica de Santa Maria Arequipa Peru

**Keywords:** natural language processing, clinical text, deep learning, long short-term memory, contextual information

## Abstract

**Background:**

Negation and speculation are critical elements in natural language processing (NLP)-related tasks, such as information extraction, as these phenomena change the truth value of a proposition. In the clinical narrative that is informal, these linguistic facts are used extensively with the objective of indicating hypotheses, impressions, or negative findings. Previous state-of-the-art approaches addressed negation and speculation detection tasks using rule-based methods, but in the last few years, models based on machine learning and deep learning exploiting morphological, syntactic, and semantic features represented as spare and dense vectors have emerged. However, although such methods of named entity recognition (NER) employ a broad set of features, they are limited to existing pretrained models for a specific domain or language.

**Objective:**

As a fundamental subsystem of any information extraction pipeline, a system for cross-lingual and domain-independent negation and speculation detection was introduced with special focus on the biomedical scientific literature and clinical narrative. In this work, detection of negation and speculation was considered as a sequence-labeling task where cues and the scopes of both phenomena are recognized as a sequence of nested labels recognized in a single step.

**Methods:**

We proposed the following two approaches for negation and speculation detection: (1) bidirectional long short-term memory (Bi-LSTM) and conditional random field using character, word, and sense embeddings to deal with the extraction of semantic, syntactic, and contextual patterns and (2) bidirectional encoder representations for transformers (BERT) with fine tuning for NER.

**Results:**

The approach was evaluated for English and Spanish languages on biomedical and review text, particularly with the BioScope corpus, IULA corpus, and SFU Spanish Review corpus, with F-measures of 86.6%, 85.0%, and 88.1%, respectively, for NeuroNER and 86.4%, 80.8%, and 91.7%, respectively, for BERT.

**Conclusions:**

These results show that these architectures perform considerably better than the previous rule-based and conventional machine learning–based systems. Moreover, our analysis results show that pretrained word embedding and particularly contextualized embedding for biomedical corpora help to understand complexities inherent to biomedical text.

## Introduction

A part of clinical data is often described in unstructured free text, such as that recorded in electronic health records (EHRs), medical records, and clinical narrative, which is not analyzed. Besides, scientific literature databases collect valuable publications necessary to extract biomedical data, such as drug or protein interactions, adverse drug effects, disabilities, diseases, treatments, detection of cancer symptoms, and suicide prevention. Biomedical experts and clinicians need to access information and knowledge in their different research areas, convert research results into clinical practice, accelerate biomedical research, provide clinical decision support, and generate data and information in a structured way for downstream processing and applications, such as those specified previously [1]. However, identifying all the data in unstructured documents and translating these data to structured data can be a complex and time-consuming task. It is impossible for experts to process all the documents without tools that filter, classify, and extract information. That is why new techniques are necessary for the extraction of useful knowledge in a precise and efficient way.

**Figure 1:**
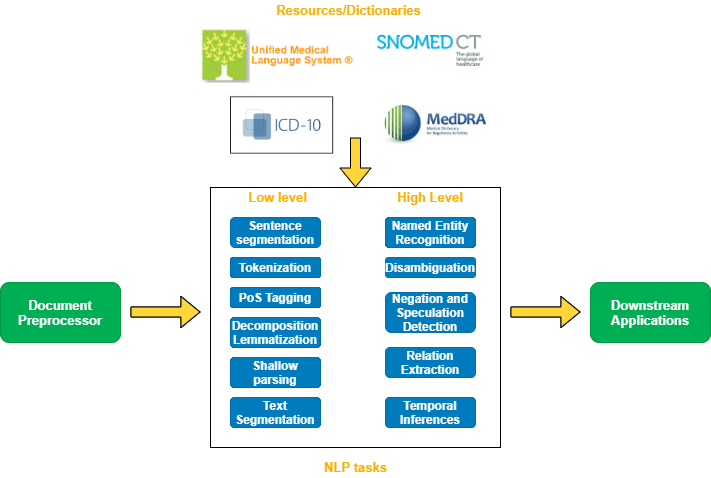
Typical information extraction pipeline. NLP: natural language processing; PoS: part of speech.

**Figure 2:**
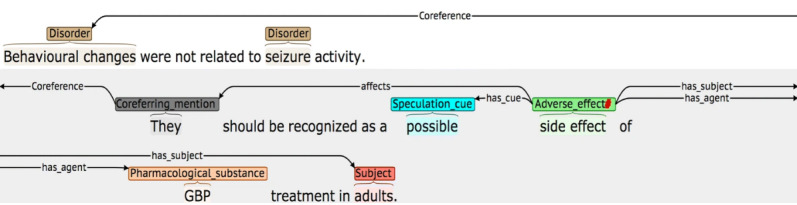
Information extraction pipeline annotation result [2].

One of the main tools currently used for text mining is natural language processing (NLP) and specifically an information extraction system. Information extraction is devoted to processing text and detecting relevant information about specific subjects (for instance, a disease of a patient in a clinical note or a carcinoma in a radiologic report). In information extraction, we can identify low-level tasks and high-level tasks (Figure 1). Low-level tasks are more feasible and affordable processing tasks, such as sentence segmentation, tokenization, and word decomposition. High-level tasks are more complex tasks because they require semantic and contextual knowledge that is provided by domain-specific resources, such as ontologies, and they involve disambiguating terms (such as abbreviations that are highly ambiguous terms) and making inferences with the extracted knowledge. These high-level tasks are named entity recognition (NER), relation extraction, and negation and speculation detection, among others (Tables 1 and 2). For example, extracting a patient’s current diagnostic information involves NER, disambiguation, negation and speculation detection, relation extraction, and temporal inference. Figure 2 provides an example of an annotation generated by a medical information extraction system [2].

**Table 1:** Natural language processing low-level tasks.

Task	Objective	Challenge
Sentence segmentation	Detection limit of a sentence.	High use of abbreviations and titles such as “mg” and “Dr” makes this task difficult.
Tokenization	Detection of words and punctuation marks.	Terms combining different types of alphanumeric characters and other signs, such as hyphens, slash, and separators (“10 mg/day” and “N-acetylcysteine”).
Part-of-speech (PoS) tagging	Assigns a PoS tag to a term.	Use of homographs and gerunds.
Decomposition/lemmatization	Word stemming by removing suffixes. Very important for concept normalization.	Many medical terms, such as “nasogastric,” need decomposition to understand the meaning of the term.
Shallow parsing	Identification of the phrases of a sentence.	Inherent complexities from the language (for instance, prepositional attachment).
Text segmentation	Division of the text into relevant parts, such as paragraphs, sections, and others.	In a clinical report, identify sections, such as patient’s history, diagnosis, treatment, etc.

**Table 2:** Natural language processing high-level tasks.

Task	Objective	Challenge
Named entity recognition	Identification and classification of concepts of interest, such as diseases, drugs, and genes.	Multitoken concepts (“acute rhinovirus bronchitis”) and short concepts (“mg”).
Disambiguation	Identification of the correct sense of a term given a specific context.	A considerable number of abbreviations with several senses, such as Pt (patient/physiotherapy) and LFT (liver function test/lung function test).
Negation and speculation detection	Inferring whether a named entity is present or absent.	They are commonly marked in the clinical narrative by words such as “not” and “without.”
Relation extraction	Identification of relationships between concepts.	Relation between a particular disease and a specific symptom or drug-drug interaction. For example, pharmacodynamic interaction between aspirin and ibuprofen (antagonistic interaction).
Temporal inferences	Given temporal expressions or temporal relationships, inferences are made about probable events in another temporal space.	The most complex task in information extraction. For example, “asbestos exposure and smoking until a particular genetic mutation occurs causes lung cancer in 1-3 years with a probability of 0.2.”

Consequently, information extraction tools must address many inherent natural language challenges, such as ambiguity, spelling variations, abbreviations, speculation, and negation. In this work, we address the negation and speculation problems. Negation and speculation expressions are extensively used both in spoken and written communications. Negation converts a proposition represented by a linguistic unit (sentence, phrase, or word) into its opposite, for instance, the existence or absence of medical conditions in a clinical narrative. It is marked by words (such as “not” and “without”), suffixes (such as “less”), or prefixes (such as “a”). Around 10% of the sentences in MEDLINE abstracts include negation phenomena [3]. The BioScope corpus contains more than 20,000 sentences, among which almost 2000 (11.4%) are negated or uncertain sentences [4]. In the general domain, the SFU ReviewSP-NEG corpus is composed of approximately 9455 sentences, among which nearly a third are negated or uncertain sentences [5]. Different works have shown the importance of dealing with negations, for instance, during the analysis of EHRs [1] or in information retrieval tasks on rare disease patient records related to Crohn disease, lupus, and NPHP1 from a clinical data warehouse [6]. In relation to speculation (or modality), both are referred to as expressing facts that are not known with certainty (such as hypotheses and conjectures). There are different types of expressions that have speculation meanings as follows: modal auxiliaries (must/should/might/may/could be), judgment verbs (suggest), evidential verbs (appear), deductive verbs (conclude), adjectives (likely), adverbs (perhaps), nouns (there is a possibility), conditional words, etc.

These phenomena have a scope, that is, affect a part of the text denoted by the presence of negation or speculation cues. Cues usually occur in the context of some assumption, which works to deny or counteract that assumption. These cues can be single words, simple phrases, or complex verb phrases, which may precede or succeed the words that are within their scope [7]. According to grammar, the scope of the negation or speculation corresponds to the totality of words affected by it. In NLP, negation or speculation cues act as operators that can change the meaning of the words in their scope. Thus, they establish what is a fact and what is not, owing to the ability to affect the truth value of a phrase or sentence [8]. However, negation detection is a complex task owing to the multiple forms in which it can appear as follows: (1) syntactic (ie, negation in sentences, clauses, and phrases that include words expressing negation, such as no/not, never/ever, and nothing), (2) lexical negation (eg, “lack of”), and (3) morphological negation (eg, illegal and impossible) [5].

Negation processing can be divided into two phases. First, keywords/cues indicating negation or speculation are detected, and second, definition of the linguistic scope of these cues is made at the sentence level. In English, negation and speculation detection is a well-studied phenomenon. However, in other languages, such as Spanish, it is an underaddressed and even more complicated task owing to the limited number of annotated corpora and the inherent complexities of the language, such as double negation (eg, the hospital will not allow no more visitors). NegEx [9], one of the most popular rule-based algorithms for negation detection in English, is a simple regular expression-based algorithm that uses negation cue words without considering the semantics of a sentence. Some recent works also exploit this algorithm for negation detection in other languages, such as French, German, and Swedish [10], Swedish [11], and Spanish [12]. Machine learning methods have been applied to cope with the negation detection task, using mainly a conditional random field (CRF) algorithm with dense vector features, such as character or word embedding [13,14]. More recently, deep learning approaches using recurrent neural networks (RNNs), convolutional neuronal networks (CNNs), and encoder-decoder models have also been exploited to solve this task [15-17].

In this work, we addressed the negation and speculation detection tasks as named entity recognition (NER) tasks that solve the identification of cues and scope of this phenomena in a single step. We present two deep learning approaches. First, we implemented two bidirectional long short-term memory (Bi-LSTM) layers with a CRF layer based on the NeuroNER model proposed previously [18]. Specifically, we extended NeuroNER by adding context information to the character and word-level information, such as part-of-speech (PoS) tags and information about overlapping or nested entities. Moreover, in this work, we used several pretrained word-embedding models as follows: (1) word2vec model (Spanish Billion Word Embeddings [19]), which was trained on the 2014 dump of Wikipedia, (2) pretrained word2vec model of word embedding trained with PubMed and PubMed Central articles [20], and (3) sense-disambiguation embedding model [21], where different word senses are represented with different sense vectors. To the best of our knowledge, no previous work has exploited a sense embedding model for the negation detection task. Finally, we implemented the bidirectional encoder representations for transformers (BERT) model with fine tuning using a BERT multilingual pretrained model.

Since the health care system has started adopting cutting-edge technologies, there is a vast amount of data collected mainly in unstructured formats, such as clinical narratives, electronic reports, and EHRs. Therefore, there is a high amount of unstructured data. All of these data involve relevant challenges for information extraction and utilization in the health care domain through various applications of NLP in health care, such as clinical trial matching [22], automated registry reporting, clinical decision support [23], and predicting health care utilization [24]. However, all these applications must deal with inherent NLP challenges, with negation and speculation detection being highly crucial owing to the abuse of negation and speculation particles in the clinical narrative and clinical records.

**Figure 3:**
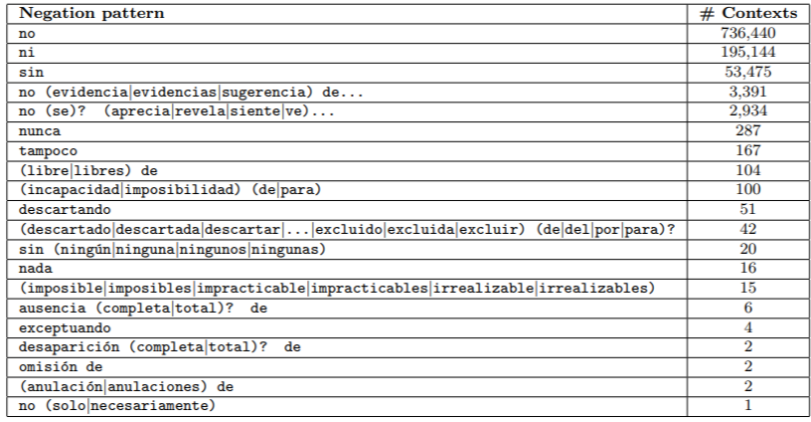
Statistics of the set of negation patterns [30].

Work in negation detection has focused on the following two subtasks: (1) cue detection to identify negation terms and (2) scope resolution to determine the coverage of a cue in a phrase or sentence. However, in previous research, negation detection has focused on the straight detection of negated entities [17]. Early negation detection work has relied on rule-based approaches. Rule-based approaches have been shown to be effective in NLP challenges. They use hand-crafted rules based on grammatical patterns and keyword matching. Some token-based systems are NegEx [25], NegFinder [26], NegHunter [27], and NegExpander [28]. DepNeg [29] uses syntactic parsing. Among rule-based approaches, the most used negation detection tool in English is NegEx [13], which employs an exact match to a list of medical entities and negation triggers (eg, “NO history of exposure” and “DENIES any nausea”). NegEx was adapted to address negation detection for other languages, such as Swedish [11], French [30], German [12], and Spanish [31]. Light et al [3] used a hand-crafted list of negation cues to identify speculation sentences in MEDLINE abstracts. Likewise, several biomedical NLP studies have used rules to identify the speculation of extracted information [32-35]. An analysis of a set of Spanish clinical notes from a hospital [36] reported some statistics of several groups of patterns considering the groups defined in the NegEx algorithm [25] as follows: morphologically negates, adverbs, prenegative phrases, postnegative phrases, and pseudonegative phrases. These patterns were applied to the data set, and only the more frequent patterns were inspected (about 100 contexts per pattern). Figure 3 shows the frequencies of the set of negation patterns in the studied corpus, where negation patterns using adverbs (“no,” “ni,” and “sin”) are the more productive patterns, followed by adverbs together with evidential and perception verbs (eg, “no se evidencia” + symptom). There are other negation words, such as “nadie” (nobody) and “negative” (negative), which do not appear in the data set.

Approaches to speculation and negation detection that exploit semisupervised or supervised machine learning models require manually labeled corpora. Medlock [37] used spare word representation features as inputs to classify sentences from biological articles (included in the molecular biology database FlyBase) as certain or uncertain based on semiautomatically collected training examples. Vincze et al [4] extended this approach [37] incorporating n-gram features and a semisupervised selection of keyword features. Morante and Daelemans [38] created a negation cue and scope detection system in biomedical text. This system identifies negation cues using the compressed decision tree (IGTREE) algorithm. It uses a meta-learner based on memory-based learning, a support vector machine, and conditional random fields (CRFs) for determining the scope of the negation. The system was evaluated on the BioScope data set [4], with an F-measure of 98.74% for cue detection and 89.15% for scope determination. Cruz et al [39] focused on negation cue detection in the BioScope corpus using the C4.5 and naive bayes algorithms, with the top F-measure of 86.8% for biomedical articles. Other studies have incorporated POS tag information [40] or different classifiers [41] that followed the two-step approach. Zou et al [42] proposed a tree kernel–based method for scope identification, based on structured syntactic parse features. The system was evaluated on the BioScope corpus, achieving a valuable improvement compared with the state-of-the-art approach, with an F-measure of 92.8% for negation detection.

In previous years, negation and speculation detection was being addressed as a sequence-labeling task. One of the most used algorithms for negation detection is CRF. White et al [43] proposed a CRF-based model with a set of lexical, structural, and syntactic features for scope detection. Kang et al [14] incorporated character-level and word-level dense representations (embeddings) in a CRF algorithm. The best F-measure was 99% for cue detection and 94% for scope detection in Chinese text, and it was concluded that embedding features can help to achieve better performance. Santiso et al [13] proposed a similar system using spare and dense word feature representations and a CRF algorithm to detect only negated entities in Spanish clinical text. The system obtained F-measures of 45.8% and 81.2% for the IxaMed-GS corpus [44] and the IULA corpus [45], respectively.

However, more recently, deep learning approaches are getting more attention, specifically RNNs and CNNs. Lazib et al [46] proposed a hybrid RNN and CNN system with a feature set of word embedding and a syntactic path (the shortest syntactic path from the candidate token to the cue in both constituency and dependency parse trees) to treat this task, and it proved to be very powerful in capturing the potential relationship between the token and the cue. Later, Lazib et al [47] proposed various RNN models to automatically find the part of the sentence affected by a negation cue. They used an automatically extracted word embedding representation of the terms as the only feature. Their Bi-LSTM model achieved an F-measure of 89.38% for the SFU review corpus [48], outperforming all previous hand-encoded feature-based approaches.

Similarly, Fancellu et al [49] used a Bi-LSTM model to solve the task of negation scope detection, and it outperformed the best result of Sem shared task 2012 [50]. Some approaches were proposed to rely on syntactic parse information to automatically extract the most relevant features [51]. Qian et al [15] designed a CNN-based model with probabilistic weighted average pooling to address speculation and negation scope detection. Evaluation of the BioScope corpus showed that their approach achieved substantial improvement. Finally, Bathia et al [17] proposed an end-to-end neural model to jointly extract entities and negations based on the hierarchical encoder-decoder NER model. The system was evaluated on the 2010 i2b2/VA challenge data set, obtaining an F-score of 90.5% for negation detection.

Motivated by the recent success of machine learning and deep learning approaches in solving various NLP issues, in this paper, we proposed the following two methods: (1) a machine and deep learning model combining two Bi-LSTM networks and a last CRF network, and (2) a BERT model with fine tuning to solve negation and speculation detection issues in multidomain text in both English and Spanish. Negation processing in the Spanish clinical narrative has been little addressed in previous years. Moreover, to the best of our knowledge, sense or context embedding has not been exploited for the negation detection task.

## Methods

### Overview

We addressed the task of negation and speculation detection as a sequence-labeling task, where we classified each token in a sentence as being part of the negation or speculation cue or negation scope. We have presented the data sets used for training, validating, and evaluating our systems. We have presented a deep network with a preprocessing step, a learning transfer phase, two recurrent neural network layers, and the last layer with a CRF classifier. Moreover, to compare our system performance, we used a baseline model based on a multilayer bidirectional transformer encoder.

### NER Architecture

**Figure 4:**
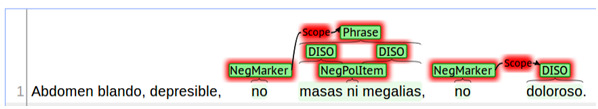
Examples of annotations in BRAT format over a sentence extracted from the IULA Spanish Clinical Record corpus (translation to English: soft, depressible abdomen, no masses or megalias, not painful).

We have address the NER task as a sequence-labeling task. In order to train our model, first, text must be preprocessed to create the input for the deep network. Sentences were split and tokenized using Spacy [52], an open-source library for advanced NLP with support for 26 languages. The output from the previous process was formatted to BRAT format [53]. BRAT is a standoff format where each line represents an annotation (such as entity, relation, and event). We used the information from the BRAT format (example in Figure 4) to annotate each token in a sentence using BMEWO-V extended tag encoding (entity tags used in Table 3), which allowed us to capture information about the sequence of tokens in the sentence.

**Table 3:** Entity tags for BMEWO-V tag encoding in the IULA Spanish Clinical Record corpus.

Entity	Tags
NegMarker^a^	B/M/E/W/V-NegMarker
NegPolItem^b^	B/M/E/W/V-NegPolItem
NegPredMarker^c^	B/M/E/W/V-NegPredMarker
PROC^d^	B/M/E/W/V-PROC
DISO^e^	B/M/E/W/V-DISO
PHRASE^f^	B/M/E/W/V-PHRASE
BODY^g^	B/M/E/W/V-BODY
SUBS^h^	B/M/E/W/V-SUBS
Others	O

^a^NegMarker: no, tampoco, sin [4].

^b^NegPolItem: ni, ninguno, ... [4].

^c^NegPredMarker: negative verbs, nouns, and adjectives [4].

^d^PROC: procedure.

^e^DISO: clinical finding.

^f^PHRASE: nonmedical text spans.

^g^BODY: body structure.

^h^SUBS: substance pharmacological/biological product.

In BMEWO-V encoding, the B tag indicates the start of an entity, the M tag represents the continuity of an entity, the E tag indicates the end of an entity, the W tag indicates a single entity, and the O tag represents other tokens that do not belong to any entity. The V tag allows representation of overlapping entities. BMEWO-V is similar to other previous encodings [54]; however, it also allows the representation of discontinuous entities and overlapping or nested entities. As a result, we obtained the sentences annotated in CoNLL-2003 format (Table 4).

**Table 4:** Tokens annotated in the ConLL-2003 format.

Token	File	Start offset	End offset	Tag	Tag
Abdomen	negation_iac_3_corr	0	7	O^a^	O
blando	negation_iac_3_corr	8	14	O	O
,	negation_iac_3_corr	14	15	O	O
depresible	negation_iac_3_corr	16	26	O	O
,	negation_iac_3_corr	26	27	O	O
no	negation_iac_3_corr	28	30	W-NegMarker^b^	W-NegMarker
masas	negation_iac_3_corr	31	36	V-Phrase^c^	W-DISO^d^
ni	negation_iac_3_corr	37	39	V-Phrase	W-NegPolIten^e^
megalias	negation_iac_3_corr	40	48	V-Phrase	W-DISO
,	negation_iac_3_corr	48	49	O	O
no	negation_iac_3_corr	50	52	W-NegMarker	W-NegMarker
doloroso	negation_iac_3_corr	53	61	W-DISO	W-DISO
.	negation_iac_3_corr	61	62	O	O

^a^O: other (no entity annotation).

^b^NegMarker: no, tampoco, sin [4].

^c^Phrase: nonmedical text spans.

^d^DISO: clinical finding.

^e^NegPolItem: ni, ninguno, ... [4].

Unlike other detection approaches that detect negation or speculation cues in the first stage and recognize the scope of both of them in the second stage (two-stage system), we proposed a one-stage approach (threaten cue entities within scope entities as nested entities, recognizing both entities [cues and scopes] in a single stage).

### Bi-LSTM CRF Model: NeuroNER Extended

**Figure 5:**
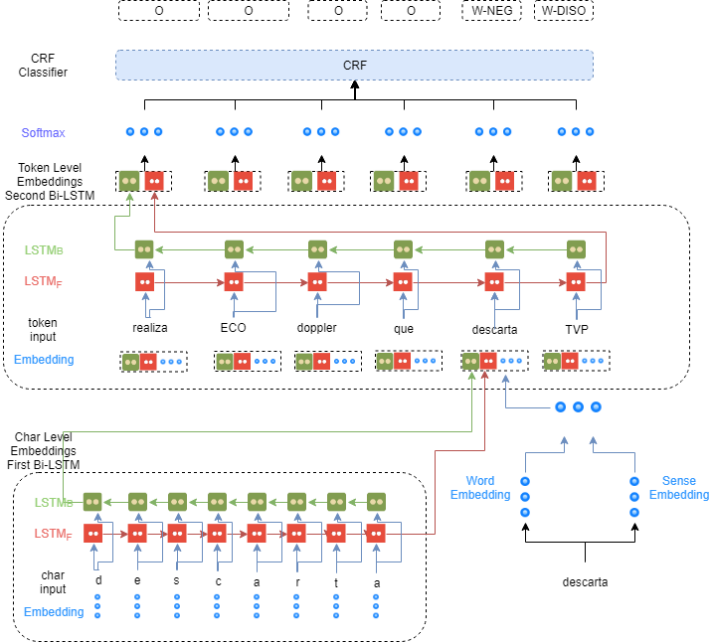
The architecture of the hybrid Bi-LSTM CRF model for negation and speculation recognition. Bi-LSTM: bidirectional long short-term memory; CRF: conditional random field.

Our proposal involves the adaption of a state-of-the-art NER model named NeuroNER [18] based on deep learning to identify entities as negation and speculation. The architecture of our model consists of an initial Bi-LSTM layer for character embedding. In the second layer, we concatenate the output of the first layer with word embedding and sense-disambiguate embedding for the second Bi-LSTM layer. Finally, the last layer uses a CRF to obtain the most suitable labels for each token. An overview of the system architecture can be seen in Figure 5.

To facilitate training of our model, we first performed a learning transfer step. Learning transfer aims to perform a task on a data set using knowledge learned from a previous data set [55]. As is shown in many studies, speech recognition [56], sentence classification [57], and NER [58] learning transfer improves generalization of the model, reduces training time on the target data set, and reduces the amount of labeled data needed to obtain high performance. We propose learning transfer as input for our model using the following two different pretrained embedding models: (1) word embedding and (2) sense-disambiguation embedding. Word embedding is an approach to represent words as vectors of real numbers, which has gained much popularity among the NLP community because it is able to capture syntactic and semantic information among words.

Although word embedding models are able to capture syntactic and semantic information, other linguistic information, such as morphological information, orthographic transcription, and POS tags, are not exploited in these models. According to a previous report [59], the use of character embedding improves learning for specific domains and is useful for morphologically rich languages (as is the case of the Spanish language). For this reason, we decided to consider the character embedding representation in our system to obtain morphological and orthographic information from words. We used a 25-feature vector to represent each character. In this way, tokens in sentences are represented by their corresponding character embeddings, which are the inputs for our Bi-LSTM network.

We used the Spanish Billion Words model [19], which is a pretrained model of word embedding trained on different text corpora written in Spanish (such as Ancora Corpus [60] and Wikipedia). Furthermore, we used a pretrained word embedding model induced from PubMed and PubMed Central texts and their combination using the word2vec tool [20]. PubMed text considers abstracts of scientific articles as of the end of September 2013, with a total of 22 million records. PubMed Central text considers full-text articles as of the end of September 2013 and constitutes a total of 600,000 articles. These resources were derived from the combination of abstracts from PubMed and full-text documents from the PubMed Central Open Access subset written in English. We also experimented with Google word2vec embedding [61] trained on 100 billion words from Google News [62].

We also integrated the sense2vec [21] model, which provides multiple embeddings for each word based on the sense of the word. This model is able to analyze the context of a word and then assign a more adequate vector for the meaning of the word. In particular, we used the Reddit Vector, a pretrained model of sense-disambiguation representation vectors introduced previously [21]. This model was trained on a collection of comments published on Reddit (corresponding to the year 2015). The details of pretrained embedding models are shown in Table 5.

**Table 5:** Details of the pretrained embedding models.

Detail	Spanish Billion Words	Google News	PubMed and PubMed Central	Reddit
Language	Spanish	English	English	Multilingual
Corpus size	1.5 billion	100 billion	6 trillion	2 billion
Vocab size	1 million	3 million	2 million	1 million
Array size	300	300	200	128
Algorithm	Skip-gram BOW	Skip-gram BOW	Skip-gram BOW	Sense2Vec

The output of the first layer was concatenated with word embedding and sense-disambiguation embedding obtained from pretrained models for each token in a given input sentence. This concatenation of features was the input for the second Bi-LSTM layer. The goal of the second layer was to obtain a sequence of probabilities corresponding to each label of the BMEWO-V encoding format. In this way, for each input token, this layer returned six probabilities (one for each tag in BMEWO-V). The final tag should be with the highest probability for each token.

To improve the accuracy of predictions, we also used a CRF [63] model, which takes as input the label probability for each independent token from the previous layer and obtains the most probable sequence of predicted labels based on the correlations between labels and their context. Handling independent labels for each word shows sequence limitations. For example, considering the drug sequence-labeling problem, an “I-NEGATION” tag cannot be found before a “B-NEGATION” tag or an “I- NEGATION” tag cannot be found after a “B-NEGATION” tag. Finally, once tokens have been annotated with their corresponding labels in the BMEWO-V encoding format, the entity mentions must be transformed into the BRAT format. V tags, which identify nested or overlapping entities, are generated as new annotations within the scope of other mentions.

### Multilayer Bidirectional Transformer Encoder: BERT

The use of word representations from pretrained unsupervised methods is a crucial step in NER pipelines. Previous models, such as word2vec [62], Glove [64], and FastText [65], focused on context-independent word representations or word embedding. However, in the last few years, models have focused on learning context-dependent word representations, such as ELMo [66], CoVe [67], and the state-of-the-art BERT model [68], and then fine tuning these pretrained models on downstream tasks.

BERT is a context-dependent word representation model that is based on a masked language model and is pretrained using the transformer architecture [69]. BERT replaces the sequential nature of language modeling. Previous models, such as RNN (LSTM & GRU), combine two unidirectional layers (ie, Bi-LSTM), and as a replacement for the sequential approach, the BERT model employs a much faster attention-based approach. BERT is pretrained in the following two unsupervised tasks: (1) masked language modeling that predicts randomly masked words in a sequence and hence can be used for learning bidirectional representations by jointly conditioning both left and right contexts in all layers and (2) next sentence prediction to train a model that understands sentence relationships. A previous report [70] provides a detailed description of BERT.

**Figure 6:**
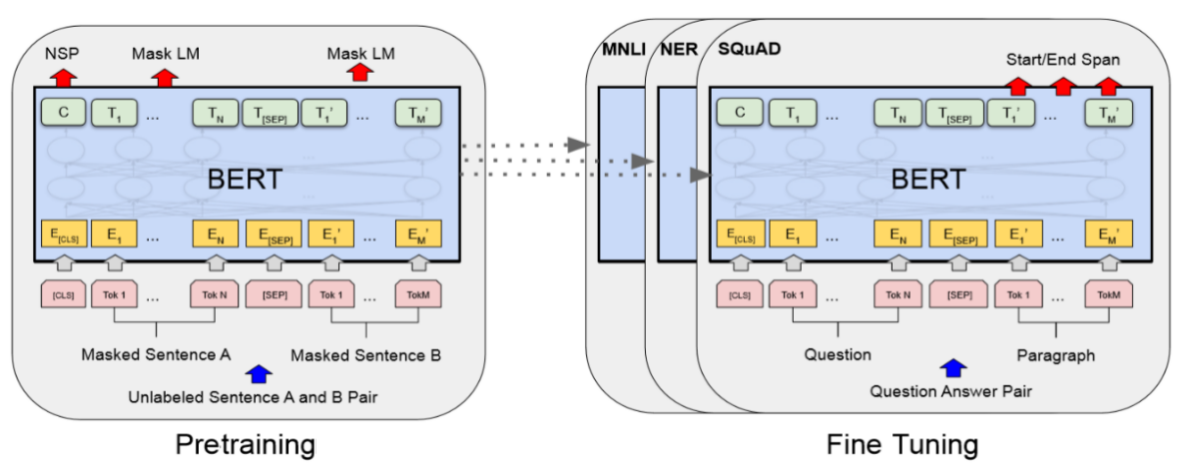
BERT pretraining and fine-tuning architecture overview [62]. BERT: bidirectional encoder representations from transformers.

Owing to the benefits of the BERT model, we adopted a pretrained BERT model with 12 transformer layers (12 layers, 768 hidden, 12 heads, 110 million parameters) and an output layer with SoftMax to perform the NER task. The transformer layer has the following two sublayers: a multihead self-attention mechanism, and a position-wise, fully connected, feed-forward network, followed by a normalization layer. An overview of the BERT architecture is presented in Figure 6.

### Data Sets

The proposed systems are evaluated for the following three data sets: (1) the BioScope corpus introduced in the CoNLL-2010 Shared Task [7] for the detection of speculation cues and their linguistic scope [4], (2) the SFU ReviewSP-NEG corpus used in Task 2 in the 2018 edition of the Workshop on Negation in Spanish (NEGES 2018) [71], and (3) the IULA Spanish Clinical Record corpus [72]. Therefore, we evaluated the proposed system in two different languages (English and Spanish) and different text types (clinical narrative, biomedical literature, and user reviews). Spanish, contrary to other languages such as English, does not have enough corpora, data sets, pretrained models, and resources. Furthermore, research on Spanish negation and speculation detection is insufficient, and this is even more in the biomedical domain. Being aware of this setback, in this particular study, we used the scarce Spanish resources available.

The BioScope corpus is a widely used and freely available resource consisting of medical and biological texts written in English annotated with speculative and negative cues and their scopes. BioScope includes the following three different subcorpora: (1) clinical free texts (clinical radiology records), (2) full biological papers from Flybase and the BMC Bioinformatics website, and (3) biological abstracts from the GENIA corpus [73]. The corpus statistics are shown in Table 6.

**Table 6:** BioScope corpus details.

Variable		Abstracts	Full papers	Clinical narratives
**Total**				
	Number of documents	1954	9	1273
	Number of sentences	6383	2624	11,872
**Speculation**				
	Number of sentences	2101	519	855
	Number of scopes	2659	672	1112
**Negation**				
	Number of sentences	1597	339	865
	Number of scopes	1719	376	870

Concerning negation and speculation, the CoNNLL-2010 Shared Tasks divide the BioScope data set into three subtasks. The first two subtasks are as follows: (1) Task 1B sentence speculation detection for biological abstracts and full articles and (2) Task 1W sentence speculation detection for paragraphs from Wikipedia, possibly containing weasel information. Both tasks consist of a binary classification problem for detecting speculation cues and speculation at the sentence level and the final task (Task 2), which aims the in-sentence hedge scope to distinguish uncertain information from facts in general and biomedical domains. The BioScope corpus includes a different data set for each subtask. Detailed information about these data sets can be seen in Table 7.

**Table 7:** BioScope subtask data sets.

Task and subset		Number of documents	Number of sentences		Number of cues		Number of scopes
**Task 1B**							
	Training	966	10,806	2540		N/A^a^	
	Validation	316	3735	836		N/A	
	Testing	15	5003	N/A		N/A	
**Task 1W**							
	Training	1646	8343	2363		N/A	
	Validation	540	2768	770		N/A	
	Testing	2346	9634	N/A		N/A	
**Task 2**							
	Training	966	11,009	2556		2519	
	Validation	316	3533	820		808	
	Testing	15	5003	N/A		N/A	

^a^N/A: not applicable.

The IULA Spanish Clinical Record corpus consists of 300 manually annotated and anonymized clinical records from several services of one of the main hospitals in Barcelona. These clinical records are written in Spanish. The corpus contains annotations on syntactic and lexical negation markers and their respective scopes. Morphological negation was excluded. There are 3194 sentences, and of these, 1093 (34.22%) were annotated with negation cues. IULA Spanish Clinical Record corpus details and its entity distribution can be found in Tables 8 and 9, respectively.

**Table 8:** IULA Spanish Clinical Record corpus details.

Item	Clinical narrative, n
Documents	300
Sentences	3194
Annotated sentences	1093
Negated entities	1456

**Table 9:** IULA Spanish Clinical Record corpus entity distribution.

Entity	Total, n
NegMarker^a^	1007
NegPredMarker^b^	86
NegPolItem^c^	114
BODY^d^	7
SUBS^e^	14
DISO^f^	1064
PROC^g^	93
Phrase^h^	278

^a^NegMarker: no, tampoco, sin [4].

^b^NegPredMarker: negative verbs, nouns, and adjectives [4].

^c^NegPolItem: ni, ninguno, ... [4].

^d^BODY: body structure.

^e^SUBS: substance pharmacological/biological product.

^f^DISO: clinical finding.

^g^PROC: procedure.

^h^PHRASE: nonmedical text spans.

To the best of our knowledge, the IULA Spanish Clinical Record corpus has not been used in any task or challenge. Therefore, we randomly split the data set into training, validation, and testing data sets. Details about the data sets can be seen in Table 10.

**Table 10:** IULA Spanish Clinical Record data sets.

Subset	Number of sentences	Number of entities
Training	1774	2839
Validation	701	924
Testing	719	920

The SFU ReviewSP-NEG corpus is the first Spanish corpus that includes event negation as part of the annotation scheme, as well as the annotation of discontinuous negation markers. Moreover, it is the first corpus where the negation scope is annotated. The corpus also includes syntactic negation, scope, and focus. However, neither lexical nor morphological negation is included. Annotations on the event and on how negation affects the polarity of the words within its scope are also included. The Spanish SFU Review corpus consists of 400 reviews from the Ciao website [74] from the following eight different domains: cars, hotels, washing machines, books, phones, music, computers, and movies. It is composed of 9455 sentences, and of these, 3022 (31.97%) contain at least one negation cue. SFU ReviewSP-NEG corpus text distribution can be found in Table 11. The SFU ReviewSP-NEG corpus was used in Task 2 of NEGES 2018 for identifying negation cues in Spanish. The data set was randomly divided into training, validation, and testing data sets. Details about the data sets can be seen in Table 12.

**Table 11:** SFU ReviewSP-NEG corpus details.

Item	Reviews, n
Comments	400
Sentences	9455
Annotated sentences	3022
Negated entities	3941

**Table 12:** SFU ReviewSP-NEG data sets.

Subset	Reviews, n	Sentences, n	Negated entities, n
Training	264	1774	606
Validation	56	701	209
Testing	80	719	285

Negation cues and scope are annotated in each corpus (the IULA corpus does not include the subject within the scope). Regarding the negation in coordinated structures, the corpora also show differences. In the SFU ReviewSP-NEG corpus, a distinction is made between the coordinated negative structures. Each negation cue is independent and has its own scope. Moreover, the scopes of those negative structures with discontinuous negation cues consider the whole coordination. The IULA Spanish Clinical Record always includes coordination within the scope. Furthermore, we found that double negation (eg, “No síntoma de disnea NI dolor torácico” [No symptoms of dyspnea or chest pain]) and negation locutions, which are multiword expressions that express negation (eg, “con AUSENCIA DE vasoespasmo” [with absence of vasospasm]) were only addressed in the SFU ReviewSP-NEG corpus. Additionally, speculative expressions and uncertain annotations (eg, “Earths and clays MAY have provided prehistoric peoples”) were only addressed in the BioScope corpus.

## Results

We evaluated the negation detection system using the training, validation, and testing data sets provided by the task organizers for the CoNLL-2010 Shared Task (BioScope) and for Task 2 of NEGES 2018 (SFU ReviewSP-NEG). The IULA Spanish Clinical Record corpus has not been previously applied to any task or competition. Therefore, we split the corpus randomly into training and testing data sets to evaluate the proposal in the clinical domain.

The Bi-LSTM CRF model was trained using available pretrained word and sense embedding models on general and biomedical domains for Spanish, English, and multilingual texts. We evaluated the use of multidomain and multilanguage pretrained embedding models (general domain word and sense embeddings and multilanguage NLP tools) on the BioScope Task 1W data sets (biomedical domain and English text), with a precision, recall, and F-score of 86.2%, 87%, and 86.6%, respectively. Based on our experiments, we found that the use of specific domain (biomedical) and specific language (English) embeddings highly improved the negation and speculation detection task (Table 13). Moreover, to evaluate the performance impact, we evaluated each of our proposed features and made comparisons with base NeuroNER implementation with PubMed and PubMed Central word embeddings on the BioScope Task 1W test data set. As shown in Table 14, sense feature representation and the BIOES-V tag encoding format improved each token representation, which implies that features play different roles in capturing token-level features for NER tasks, thus making improvements in their combination.

**Table 13:** Pretrained word embedding model evaluation on the BioScope Task 1W test data set.

Name–embedding	Precision (%)	Recall (%)	F-score (%)
NeuroNER–Google News	78.3	80.4	79.3
NeuroNER–PubMed and PubMed Central	80.8	82.1	81.4
NeuroNER Extended–Google News	80.2	83.2	81.7
NeuroNER Extended–PubMed and PubMed Central	86.2	87.0	86.6

**Table 14:** Feature evaluation on the BioScope Task 1W test data set.

Name–feature	Precision (%)	Recall (%)	F-score (%)
NeuroNER–Base	78.3	80.4	81.4
NeuroNER–Sense	84.7	86.2	85.4
NeuroNER–BIOES-V	81.7	83.5	82.6
NeuroNER–Sense and BIOES-V	86.2	87.0	86.6

Moreover, we used the pretrained BERT multilingual general domain model with 12 transformer layers (12 layers, 768 hidden, 12 heads, 110 million parameters) trained on the general domain Wikipedia and Bookcorpus corpora, and fine-tuned for NER using a single output layer based on the representations from its last layer to compute only token-level BIOES-V probabilities. BERT directly learns WordPiece embeddings during the pretraining and fine-tuning steps.

Precision, recall, and the F-score were used to evaluate the performance of our system. The parameters of the sets and the hyperparameters for our Bi-LSTM CRF model are summarized in Table 15. The hyperparameters were optimized on each validation data set.

**Table 15:** NeuroNER system hyperparameters for each task.

Parameter	BioScope	IULA	SFU ReviewSP-NEG
Language	English	Spanish	Spanish
Pretrained word embedding	PubMed and PubMed Central + Reddit	Spanish Billion Words + Reddit	Spanish Billion Words + Reddit
Sense-disambiguation embedding dimension	128	128	128
Word embedding dimension	200	300	300
Character embedding dimension	50	50	50
Hidden layers dimension (for each LSTM)	100	100	100
Learning method	Stochastic gradient descent	Stochastic gradient descent	Stochastic gradient descent
Dropout rate	0.5	0.5	0.5
Learning rate	0.005	0.005	0.005
Epochs	100	100	100

The CoNLL-2010 Shared Task [75] considers two different evaluation criteria. Task 1 is made at the sentence level, and cue annotations in the sentence are not considered. However, it is optionally evaluated. The F-measure of the speculation class is employed as the chief evaluation metric. Task 2 involves the annotation of “cue” + “xcope” tags in sentences. The scope-level F-measure is used as the chief metric where true positives are scopes that match the gold standard clue words and gold standard scope boundaries assigned to the clue words.

Tables 16 to 20 compare the results obtained by the participating systems in the CoNLL-2010 Shared Task and our deep learning approach using pretrained embedding models and the BMEWO-V encoding format. Our extended version of NeuroNER achieved similar results to the best work presented in this task. In particular, our system achieved the highest precision (83.2%), with lower recall.

For subtask 1 (identification speculation at the sentence level and cue annotations), our system obtained the top F-score for speculation and cue detection (see Tables 16 to 18).

**Table 16:** Task 1B Wikipedia sentence-level speculation detection (BioScope).

Name	Precision (%)	Recall (%)	F-score (%)
Georgescul [76]	72.0	51.7	60.2
Ji et al [77]	62.7	55.3	58.7
Chen et al [78]	68.0	49.7	57.4
BERT	83.7	48.5	61.4
NeuroNER Extended	83.2	41.0	54.9

**Table 17:** Task 1B Wikipedia cue-level detection (BioScope).

Name	Precision (%)	Recall (%)	F-score (%)
Tang et al [79]	63.0	25.7	36.5
Li et al [80]	76.1	21.6	33.7
Özgür et al [81]	28.9	14.7	19.5
BERT	63.7	33.2	43.6
NeuroNER Extended	63.0	25.7	36.5

**Table 18:** Task 1W biological sentence-level speculation detection (BioScope).

Name	Precision (%)	Recall (%)	F-score (%)
Tang et al [79]	85.0	87.7	86.4
Zhou et al [82]	86.5	85.1	85.8
Li et al [80]	90.4	81.0	85.4
BERT	85.5	87.3	86.4
NeuroNER Extended	86.2	87.0	86.6

**Table 19:** Task 1W biological cue-level detection (BioScope).

Name	Precision (%)	Recall (%)	F-score (%)
Tang et al [79]	81.7	81.0	81.3
Zhou et al [82]	83.1	78.8	80.9
Li et al [80]	87.4	73.4	79.8
BERT	80.7	79.5	80.1
NeuroNER Extended	81.4	79.2	80.3

**Table 20:** Task 2 cue-level detection and scope determination (BioScope).

Name	Precision (%)	Recall (%)	F-score (%)
Morante et al [83]	59.6	55.2	57.3
Rei et al [6]	56.7	54.6	55.6
Velldal et al [84]	56.7	54.0	55.3
BERT	46.1	55.6	50.4
NeuroNER Extended	50.4	40.3	44.8

Table 21 shows the results for the IULA corpus. Furthermore, we compared our results with the work presented previously [85]. We used the evaluation criteria presented in this work; however, the subsets were different. As can be seen, our system outperformed the results obtained previously [85], with a difference of nearly 4 points for the F-measure.

**Table 21:** Results of cue level and scope detection for the IULA Clinical Record data set.

Name	Precision (%)	Recall (%)	F-score (%)
Santiso et al [85]	79.1	83.5	81.2
BERT	77.8	84.3	80.8
NeuroNER Extended	84.2	85.9	85.0

The NEGES 2018 Task 2 negation cue detection uses the evaluation script proposed in the SEM 2012 Shared Task–Resolving the Scope and Focus of Negation [50]. Table 22 shows the results for the different domains included in the data set. It can be observed that the F-score was always over 80%. We compared our results with the participating systems presented in this task. A detailed description of the evaluation has been provided previously [71]. As can be seen in Table 23, our system outperformed the rest of the participating systems.

Furthermore, we compared NeuroNER Extended and BERT implementations in terms of resources and time consumption on the IULA Clinical Record training and validation subsets. As shown in Table 24, the training time was slightly higher in NeuroNER Extended. However, training implies the generation of character and token level embeddings, unlike the BERT implementation that obtains word vector representations directly from the pretrained model. In terms of hardware resource consumption, we found that BERT implementation had a high use of resources, especially RAM and GPU.

**Table 22:** NeuroNER Extended results of negation detection for the SFU ReviewSP-NEG data set.

Domain	Precision (%)	Recall (%)	F-score (%)
Cars	87.5	74.47	80.46
Hotels	95.92	77.05	85.46
Washing machines	94.44	75.56	83.95
Books	95.45	87.5	91.3
Phones	97.06	90.83	93.84
Music	92.31	92.31	92.31
Computers	95.45	80.77	87.5
Movies	95.88	84.55	89.86

**Table 23:** Results of negation cues and scope detection for the SFU ReviewSP-NEG data set.

Name	Precision (%)	Recall (%)	F-score (%)
Fabregat et al [86]	79.5	59.6	68.0
Loharja et al [87]	79.1	83.5	81.2
BERT	92.6	90.8	91.7
NeuroNER Extended	94.3	82.9	88.1

**Table 24:** Training parameters for the deep learning models.

Training parameter	Specifications	NeuroNER Extended	BERT
CPU	Intel Core i7 7700 at 3.60 GHz	50%	30%
RAM	16 GB DDR4	40%	80%
GPU	GeForce RTX 2060 SUPER 16 RAM	40%	80%
Training time	Minutes	15 min	13 min

## Discussion

### Principal Findings

We used different pretrained models and investigated their effects on performance. For NeuroNER Extended, we used general and domain-specific pretrained word embedding models, and likewise, we used pretrained multilanguage and language-specific models. We found that the use of specific domain (biomedical) and specific language pretrained models highly improved the negation and speculation detection. Moreover, to the best of our knowledge, there is no pretrained biomedical Spanish model for context-dependent word representations (pretrained BERT). The low performance of the BERT model is mainly attributed to the use of a general domain and multilingual pretrained model. However, the BERT model outperformed the NeuroNER Extended model and other state-of-the-art approaches in general domain data sets, such as SFU ReviewSP-NEG, and the specific domain BioScope (Task 1B data set corpus obtained from Wikipedia text).

Moreover, we presented the analysis of the most frequent false negatives and false positives for negation and speculation cues and scope detection. Negation and speculation cues, such as “would,” “apenas” (“barely”), “ni” (“neither” or “nor”), “except,” “could,” “idea,” “notion,” and “may,” are half of the time labeled as negation and speculation cues. This ambiguity led our system to classify some tokens as false positive or inversely as false negative, causing a drop in performance. Furthermore, some multitoken negation and speculation cues, such as “ni siquiera” (“not even”), “ni tan siquiera” (“not even”), “ni si quiera” (“not even”), and “en ningún momento” (“not at any moment”), are sometimes labeled as a single token word (ie, “ni_siquiera,” “ni_tan_siquiera,” “ni_si_quiera,” and “en_ningún_momento”), and some others are labeled as multitoken cues. Long multitoken negation and speculation cues, such as “remains to be determined” and “raising the intriguing possibility,” are not detected or partially matched. This proves that shorter sentences, with shorter scopes and shorter negation and speculation cues, are easier to process. A longer sentence has a more complex syntactic structure and is tougher to be processed by the system. It should be noted that clinical text is undoubtedly distinct from biomedical text. It is characterized by short sentences (usually phrases) and misspellings, with abuse of negation particles and abbreviations, among other important features.

Furthermore, in the context of real medical applications, negation and speculation detection is a fundamental task in any information extraction system. For instance, in cohort selections for a clinical trial, patients with a specific condition are required, and it is essential to know if a term representing a disease or any other feature is negated or not in a clinical note in order to get the right answer to the query (Is the variable V valid for patient P?). An additional example would be the detection of adverse drug reactions, that is, the extraction of causal relations between drugs and diseases. It is a crucial step to discard the absence of adverse drug reactions early and thus prevent medical applications from analyzing them or providing wrong information.

### Conclusions

In this work, we proposed a system for the detection of negated entities, negation cues, negation scope, and speculation in multidomain text in English and Spanish. We addressed the speculation and negation detection task as a sequence-labeling task. Although previous studies have already applied deep learning to this task, our approach is the first to exploit sense embedding as the input of the deep network. In a sense embedding model, each meaning word is represented with a different vector. Therefore, sense embedding models can help to solve ambiguity, which is one of the most critical challenges in NLP.

Our experiments show that the use of dense representation of words (word-level embedding, character-level embedding, and sense embedding) provides good results in detecting negated entities, negation cues, and negation scope determination. Compared with previous work, our system achieved an F-score performance of over 85%, outperforming most current state-of-the-art methods for negation and speculation detection. Moreover, our work is one of the few that addressed the task for Spanish text and different domains using context-independent and context-dependent pretrained models.

In future work, we plan to test whether other supervised classifiers, such as Markov random fields and optimum path forest, would obtain more benefits from dense vector representation. That is to say, we would use the same continuous representations with the Markov random fields and optimum path forest classifiers. Moreover, we plan to train word context-dependent and independent embeddings obtained from multiple Spanish biomedical corpora to enhance word representations using different models, such as FastText and pretrained BERT. Furthermore, we plan to explore different models for embeddings that combine in a single representation not only words but also semantic information contained in domain-specific resources, such as UMLS [88] and SNOMED-CT [89].

## Abbreviations

BERT: bidirectional encoder representations from transformers
Bi-LSTM: bidirectional long short-term memory
CNN: convolutional neural network
CRF: conditional random field
NER: named entity recognition
NLP: natural language processing
PoS: part of speech
RNN: recurrent neural network
